# Supplementary figures and images for: Impact of COVID-19 on health services utilization in mainland China and its different regions based on S-ARIMA predictions
Source: PLOS Glob Public Health. 2023 Jan 6;3(1):e0001044. doi: 10.1371/journal.pgph.0001044 (PMC10021243; doi:10.1371/journal.pgph.0001044)

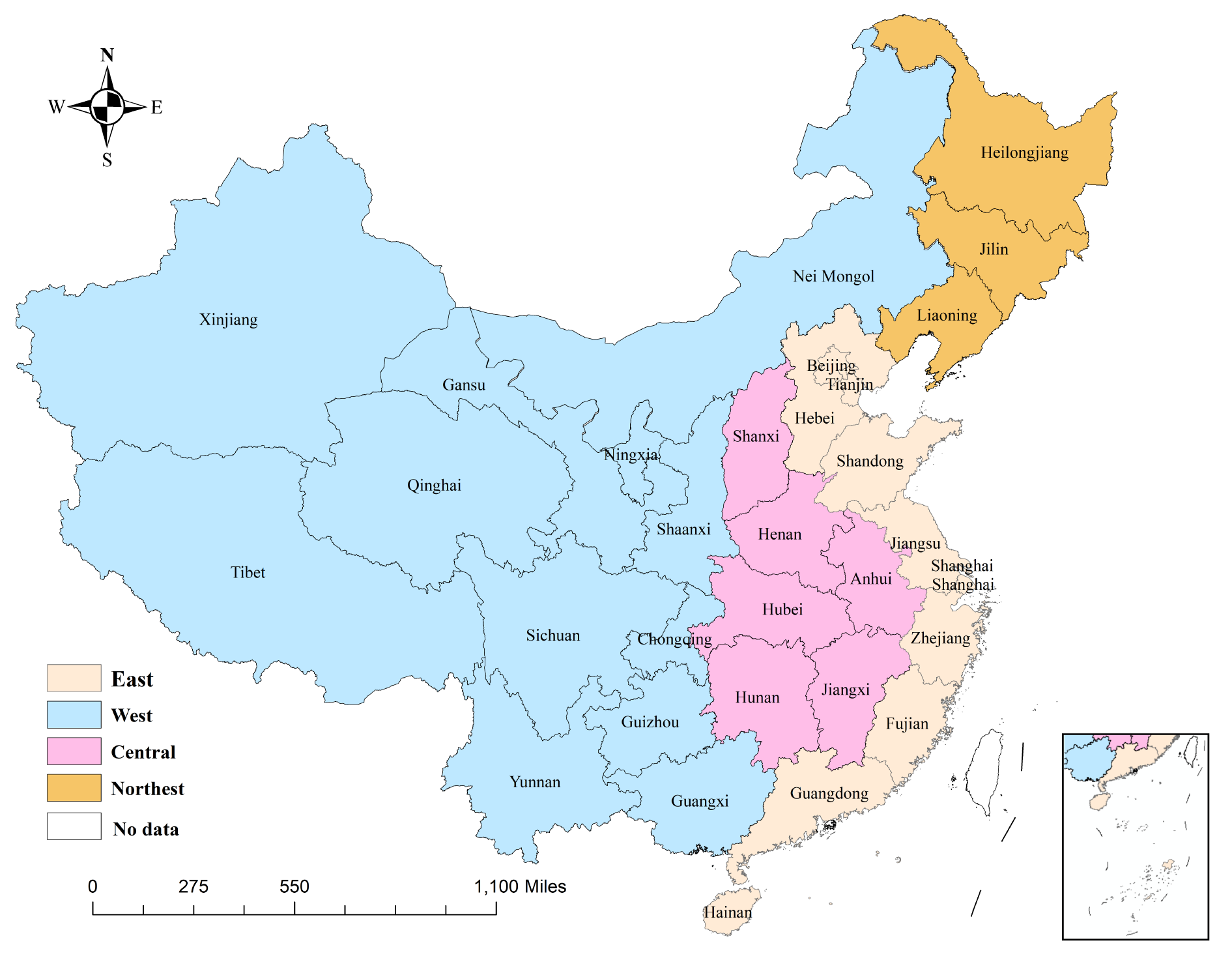

Supplement: S1 Fig — The map demonstrates geographical locations of 31 provincial administrative units in mainland China. Different background colors represent different regions. Maps were created using ArcGIS by ESRI version 10.8 (http://www.esri.com) [21]. Base map sources were at http://bzdt.ch.mnr.gov.cn/ and https://dataverse.harvard.edu/dataset.xhtml?persistentId=doi:10.7910/DVN/DBJ3BX. External data utilized in the creation of this figure from the Statistical Information Center of the National Health Commission of China (http://www.nhc.gov.cn/mohwsbwstjxxzx/s2906/new_list.shtml). (TIF) [file pgph.0001044.s001.tif]
