# Supplementary material for: Impact of COVID-19 on health services utilization in mainland China and its different regions based on S-ARIMA predictions
Source: PLOS Glob Public Health. 2023 Jan 6;3(1):e0001044. doi: 10.1371/journal.pgph.0001044 (PMC10021243; doi:10.1371/journal.pgph.0001044)
Supplement: S1 Table — No prediction and no data. AR, D, MA, SAR, S and SMA are respectively represent the S-ARIMA model specific orders of p, D, q, ps, Ds and qs. AIC and BIC respectively are Akaike Information Criterion and Bayesian Information Criterion of this model. F-S: ratio of the slope of the least binomial regression of the original and predicted series to the slope of the least binomial regression of the original series. P-S: ratio of the slope of the least binomial regression of the predicted series to the slope of the least binomial regression of the original series. (DOCX) [file pgph.0001044.s002.docx]

| **Region/Province** | | | **ARIMA** | | | **Seasonality** | | | **AIC** | **BIC** | **F-S** | **P-S** |
| --- | --- | --- | --- | --- | --- | --- | --- | --- | --- | --- | --- | --- |
|  |  |  | **AR** | **D** | **MA** | **SAR** | **S** | **SMA** |  |  |  |  |
| China | | | 1 | 1 | 1 | 11 | 11 | 11 | -308.15 | -301.88 | 1.09 | 0.62 |
|  | Central | | 1 | 1 | 1 | 11 | 11 | 11 | -323.01 | -316.74 | 1.10 | 0.38 |
|  |  | Anhui | 3 | 1 | 3 | 11 | 11 | 11 | -275.30 | -265.85 | 1.11 | 1.66 |
|  |  | Henan | 0 | 1 | 1 | 11 | 11 | 11 | -337.72 | -332.27 | 1.26 | 1.74 |
|  |  | Hubei | 1 | 1 | 1 | 11 | 11 | 11 | -289.11 | -283.14 | 0.51 | -1.67 |
|  |  | Hunan | 1 | 1 | 1 | 11 | 11 | 11 | -344.77 | -338.50 | 0.75 | 0.08 |
|  |  | Jiangxi | 2 | 1 | 1 | 11 | 11 | 11 | -342.87 | -335.90 | 1.03 | 0.97 |
|  |  | Shanxi | 2 | 1 | 3 | 11 | 11 | 11 | -310.88 | -297.98 | 0.48 | -1.37 |
|  | East | | 2 | 1 | 3 | 11 | 11 | 11 | -281.04 | -272.08 | 0.71 | -0.80 |
|  |  | Beijing | 1 | 1 | 1 | 11 | 11 | 11 | -179.79 | -173.52 | -0.89 | -9.62 |
|  |  | Fujian | 2 | 1 | 2 | 11 | 11 | 11 | -293.89 | -285.92 | 0.52 | -1.97 |
|  |  | Guangdong | 1 | 1 | 1 | 11 | 11 | 11 | -255.74 | -249.47 | 1.50 | 1.13 |
|  |  | Hainan | 3 | 1 | 2 | 11 | 11 | 11 | -275.20 | -275.20 | 0.89 | -0.05 |
|  |  | Hebei | 2 | 1 | 1 | 11 | 11 | 11 | -325.11 | -318.14 | 0.93 | 0.11 |
|  |  | Jiangsu | 2 | 1 | 1 | 11 | 11 | 11 | -252.75 | -245.78 | 0.84 | -1.58 |
|  |  | Shandong | 1 | 1 | 2 | 11 | 11 | 11 | -300.88 | -293.57 | 0.69 | -0.48 |
|  |  | Shanghai | 1 | 1 | 2 | 11 | 11 | 0 | -166.95 | -160.68 | 0.72 | 0.94 |
|  |  | Zhejiang | 0 | 1 | 1 | 11 | 11 | 11 | -243.19 | -237.73 | 0.53 | -1.51 |
|  |  | Tianjin | 2 | 1 | 2 | 0 | 11 | 0 | -202.05 | -193.45 | 0.48 | 0.17 |
|  | Northeast | | 2 | 1 | 3 | 11 | 11 | 11 | -307.88 | -298.91 | 1.27 | 0.54 |
|  |  | Heilongjiang | 2 | 1 | 2 | 11 | 11 | 11 | -335.83 | -327.86 | 1.21 | -1.25 |
|  |  | Jilin | 0 | 1 | 1 | 11 | 11 | 11 | -301.66 | -296.21 | 1.37 | 2.27 |
|  |  | Liaoning | 2 | 1 | 2 | 11 | 11 | 11 | -294.04 | -286.08 | 0.28 | -0.90 |
|  | West | | 1 | 1 | 1 | 11 | 11 | 11 | -332.06 | -325.79 | 1.09 | 1.30 |
|  |  | Chongqing | 0 | 1 | 1 | 11 | 11 | 11 | -316.59 | -311.14 | 1.01 | 1.33 |
|  |  | Gansu | 1 | 1 | 1 | 11 | 11 | 11 | -306.51 | -300.24 | 1.63 | 3.65 |
|  |  | Guangxi | 2 | 1 | 2 | 11 | 11 | 11 | -315.36 | -308.39 | 1.03 | 0.35 |
|  |  | Guizhou | 3 | 1 | 4 | 11 | 11 | 11 | -343.23 | -332.84 | 0.82 | 0.48 |
|  |  | Ningxia | 3 | 1 | 2 | 11 | 11 | 11 | -270.64 | -262.14 | 0.86 | -0.11 |
|  |  | Qinghai | 1 | 1 | 2 | 11 | 11 | 11 | -212.66 | -205.35 | 0.84 | 2.18 |
|  |  | Shaanxi | 2 | 1 | 1 | 11 | 11 | 11 | -298.68 | -291.71 | 1.09 | 1.24 |
|  |  | Sichuan | 1 | 1 | 1 | 11 | 11 | 11 | -309.02 | -302.76 | 1.09 | 1.29 |
|  |  | Tibet | - | - | - | - | - | - | - | - | - | - |
|  |  | Xinjiang | 3 | 1 | 3 | 11 | 11 | 11 | -287.15 | -277.70 | 3.15 | 40.69 |
|  |  | Yunnan | 1 | 1 | 2 | 11 | 11 | 11 | -336.17 | -328.85 | 0.86 | 0.80 |
|  |  | Nei Mongol | 0 | 1 | 1 | 11 | 11 | 11 | -285.05 | -279.60 | 1.24 | 2.11 |
