# Supplementary material for: Impact of COVID-19 on health services utilization in mainland China and its different regions based on S-ARIMA predictions
Source: PLOS Glob Public Health. 2023 Jan 6;3(1):e0001044. doi: 10.1371/journal.pgph.0001044 (PMC10021243; doi:10.1371/journal.pgph.0001044)
Supplement: Attachment — Submitted filename: Response to Reviewers.docx [file pgph.0001044.s003.docx]

Dear Dr. Lorena G Barberia,

Thank you very much for giving us the opportunity to submit a revised version of the manuscript " *Impact of COVID-19 on health services utilization in mainland China and its different regions based on S-ARIMA predictions (****#****PGPH-D-22-00696)*" for publication in *PLOS Global Public Health*. We appreciate the time and effort that you and the reviewers dedicated to providing feedback on our manuscript and are grateful for the insightful comments and suggestions to our paper. Please see below, in blue, for a point-by-point response to the reviewers’ comments and suggestions. The manuscript has also been revised according to reviewers’ comments and suggestions with tracked changes.

**Responses to reviewer 1:**
**Q1**. On page 6, the authors say: "Since OEH visits always accounts for the largest proportion of health services utilization in mainland China, and only regional variations in OEH visits per capita was observed based on our exploratory analyses (see section 3.1 in the Results), we merely took interest in the impacts on OEH visits per capita in our later analyses."

This explanation makes it unclear why the authors decided to focus only on OEH visits. I encourage them to explain this point further.

a. As the authors say, OEH visits account for the most significant proportion of utilization. To corroborate this point, please present data on the percentage of health services utilization for each of the four measures: OEH, OET, IH, and IT.

Response: We thank the reviewer for the suggestion on enriching the research context. In the time frame of this study, the sum of OEH, OET, IH and IT visits per capita account for 75.66%, 3.92%, 19.74%, and 0.68% of the total health services utilization, respectively. We have added this information in the Methods section (page 5, line 105).

In addition, the regional differences in visits per capita for the four measures were shown in **Figure R1** below. It showed that the regional heterogeneity of OEH visits per capita was more pronounced than the other three indicators.

**Figure R1. Regional differences in visits per capita for OEH, OET, IH, and IT in mainland China from 2016 to 2020.**

b. I did not find section 3.1 in the Results section. Please, make sure it is in the actual manuscript.

Response: We are sorry for this mistake. Section 3.1 refers to "*3.1 Overview of four types of health services utilization per capita in China*" in the Results section of the manuscript (page 9, line 179).

c. From what I understood from this paragraph, regional variations were only observed in OEH visits. What could explain that such regional differences were not found in OET, IH, and IT? Please, discuss this issue in the "Discussion" section.

Response: We thank the reviewer for this valuable suggestion. We have added an explanation in the Discussion section (page 15, line 325-335) as follows. We added the content in the Discussion section as follows:

"In the last decade, China's urbanization rate has raised from 46.6% to 60.6%^[1, 2]^, with a continuous increase in the number of hospitals and a consequent decrease in the amount of township health centers^[3]^. Else, deficient health workforce and unadvanced medical equipment in township health centers made people prefer hospitals when seeking for health services. Therefore, hospitals serve as the major provider of health services in mainland China. Different from hospitals, township health centers provide routine chronic medication and ailment treatment to a relatively stable number of rural residents. So, the volume of health services utilization in township health centers (i.e., OET and IT) is insusceptible to the pandemic. The possible reason why inpatient utilization rate (i.e., IH and IT) was less flexible during the pandemic could be that, patients who are advised to be hospitalized, always suffer more severe diseases or require more urgent clinical care than normal outpatients. They are less likely to delay or cancel hospitalization due to difficulties correlated with COVID-19. "

d. It should be "account" instead of "accounts" in the sentence "Since OEH visits always accounts (...)".

Response: Thanks for the correction, and we have corrected the grammar error (page 5, line 105).

**Q2**. On page 16, the authors say: "In addition, for Liaoning, Shanxi and Xinjiang, the accumulative impacts were negative, conflicted with our commonsense, indicating that the S-ARIMA model may be not suitable for the three provinces."

Given that the estimated S-ARIMA model did not work well for these three provinces, did the authors try other S-ARIMA specifications for those three provinces? Please, present the results for the alternative specifications for these cases.

Response: We thank the reviewer for this critical comment. Actually, we had tested other orders of S-ARIMA models $\left( p,D,q,p_{s},D_{s},q_{s} \right)$ and the models we chose for Liaoning, Shanxi, and Xinjiang provinces were the optimal S-ARIMA models (**Table R1** below). In the original manuscript, we presented the optimal models for every province/region in the **Appendix**.

In this study, the optimal model was judged by the following two criteria: Firstly, for a S-ARIMA model, a smaller AIC and BIC indicates stronger evidence for one model over the others (the lower the better). We selected the model that minimized the sum of AIC and BIC, and then used S-ARIMA model with above specific order to predict the series of OEH visits per capita after the pandemic (2020/02-2021/03). Secondly, we used the F-S and P-S statistics to determine the trend consistency of predicted and original series (the closer the ratio of the slope of the predicted series to the slope of the original series was to 1, the better the model predicted series could maintain the trend of the OEH visits per capita time series). Based on the above rules, and we chose S-ARIMA$(1,1,2,11,11,11)$, S- ARIMA$(2,1,2,11,11,11)$ and S-ARIMA$(3,1,3,11,11,11)$ as the optimal order model for Shanxi, Liaoning and Xinjiang, respectively.

To be clear, the current choice of S-ARIMA model did not affect the assessment of instant impacts of COVID-19 on OEH visits per capita and the accuracy of short-term forecasts was still guaranteed. Meanwhile in this study, the difference of COVID-19 impacts was mainly compared among regions, not provinces. Analyses on the regional level were not influenced as the model fits the merged data well, which did not affect the main conclusions of this study. We have also explained this in the Limitation of the manuscript (page 19, line 408-412).

**Table R1. The selection of S-ARIMA model for Shanxi, Liaoning and Xinjiang.**

| **Province** | **ARIMA** | | | **Seasonality** | | | **AIC** | **BIC** | **F-S** | **P-S** |
| --- | --- | --- | --- | --- | --- | --- | --- | --- | --- | --- |
|  | **AR** | **D** | **MA** | **SAR** | **S** | **SMA** |  |  |  |  |
| Shanxi | 2 | 1 | 3 | 11 | 11 | 11 | -310.88 | -297.98 | 0.48 | -1.37 |
|  | 2 | 1 | 2 | 11 | 11 | 11 | -306.79 | -295.32 | 0.56 | 0.40 |
|  | 3 | 2 | 3 | 11 | 11 | 11 | -308.65 | -294.98 | 0.56 | 0.40 |
|  | 1 | 1 | 2 | 0 | 0 | 0 | -267.46 | -258.65 | 1.01 | 0.90 |
|  | 1 | 1 | 1 | 11 | 11 | 11 | -314.89 | -308.63 | 0.57 | -1.07 |
|  | 1 | 1 | 2 | 11 | 11 | 11 | -317.50 | -310.19 | 0.05 | -2.72 |
|  | 2 | 2 | 2 | 11 | 11 | 11 | -303.17 | -291.96 | -0.01 | -5.48 |
|  | 3 | 1 | 2 | 11 | 11 | 11 | -304.79 | -292.18 | 0.56 | 0.40 |
|  | 1 | 2 | 1 | 11 | 11 | 11 | -301.81 | -291.77 | -0.15 | -6.43 |
|  | 2 | 1 | 1 | 11 | 11 | 11 | -308.67 | -298.63 | 0.61 | 0.59 |
|  | 1 | 1 | 2 | 22 | 0 | 11 | -315.50 | -307.15 | 0.05 | -2.72 |
|  | 1 | 2 | 2 | 0 | 11 | 11 | -286.12 | -275.55 | 0.91 | 0.51 |
|  | 3 | 1 | 3 | 11 | 11 | 11 | -308.96 | -294.95 | 0.46 | -1.47 |
|  | 1 | 1 | 2 | 11 | 0 | 11 | -308.53 | -298.27 | 0.50 | 0.19 |
| Liaoning | 2 | 1 | 2 | 11 | 11 | 11 | -294.04 | -286.08 | 0.28 | -0.90 |
|  | 1 | 1 | 2 | 11 | 11 | 11 | -290.50 | -283.18 | 1.06 | 1.41 |
|  | 3 | 1 | 2 | 11 | 11 | 11 | -293.18 | -284.68 | 1.03 | -0.05 |
|  | 2 | 2 | 2 | 11 | 11 | 11 | -297.07 | -289.51 | -0.89 | -13.77 |
|  | 1 | 1 | 2 | 11 | 11 | 11 | -290.50 | -283.18 | 1.06 | 1.41 |
|  | 1 | 1 | 1 | 11 | 11 | 11 | -291.39 | -285.12 | 1.15 | 1.50 |
|  | 1 | 2 | 2 | 11 | 11 | 11 | -284.59 | -277.62 | -0.20 | -9.34 |
|  | 3 | 2 | 2 | 11 | 11 | 11 | -295.91 | -287.90 | -0.83 | -12.79 |
| Xinjiang | 3 | 1 | 3 | 11 | 11 | 11 | -287.15 | -277.70 | 3.15 | 40.69 |
|  | 2 | 1 | 2 | 11 | 11 | 11 | -282.97 | -275.01 | 4.51 | 15.05 |
|  | 3 | 1 | 2 | 11 | 11 | 11 | -281.04 | -272.54 | 4.52 | 15.05 |
|  | 2 | 2 | 3 | 11 | 11 | 11 | -271.35 | -262.85 | 0.00 | -13.60 |
|  | 3 | 1 | 3 | 0 | 11 | 11 | -281.24 | -268.63 | 2.62 | -3.33 |
|  | 3 | 2 | 3 | 11 | 11 | 11 | -278.75 | -269.84 | 0.76 | -10.30 |

**Q3.** When the authors present and discuss the instant and cumulative impacts of the pandemic on health care visits, I encourage them to present the estimated impacts with the respective 95% confidence interval. Since the authors are estimating the impacts, it is crucial to show the uncertainty related to the estimates, which is missing in the manuscript.

Response: Thanks for the critical suggestion. We estimated 95% CI of per capita impacts and presented the results for the trend of regional impacts in the following **Figure R2** and the specific data of different regions and provinces in **Table R2** and **R3** below.

**Figure R2. Trend of impacts on the monthly OEH visits per capita by regions from October 2019 to March 2021, based on the prediction of S-ARIMA model.**

Note: The gray solid line (dot) indicates the actual number of monthly OEH visits per capita, while the solid red line (dot) represents the predicted number with its 95% upper and lower confidence limit in green and blue line, respectively. The height of each histogram and red horizontal lines (OEH visit PU) shows the value of the predicted number minus corresponding actual number, with its 95% upper and lower confidence limit in green (OEH visit PU) and blue (OEH visit PL) horizontal lines, respectively.

**Table R2. Estimated absolute impacts for four regions of mainland China by months.**

| **Region** | **Date** | **2020/02** | **2020/03** |  | **2021/02** | **2021/03** |
| --- | --- | --- | --- | --- | --- | --- |
| **Central** | **Predicted OEH** | 0.1892(0.1834,0.1951) | 0.1783(0.1721,0.1845) | … | 0.1553(0.1469,0.1638) | 0.2022(0.1936,0.2109) |
|  | **Visit impacts** | 0.1279(0.1220,0.1337) | 0.0591(0.0529,0.0653) | … | -0.002(-0.011,0.0054) | 0.0145(0.0059,0.0232) |
| **East** | **Predicted OEH** | 0.2287(0.2198,0.2375) | 0.2947(0.2853,0.3041) | … | 0.2246(0.2144,0.2348) | 0.2908(0.2805,0.3010) |
|  | **Visit impacts** | 0.1215(0.1126,0.1303) | 0.1188(0.1095,0.1282) | … | -0.007(-0.017,0.0029) | -0.000(-0.011,0.0094) |
| **Northeast** | **Predicted OEH** | 0.1972(0.1907,0.2036) | 0.2337(0.2265,0.2409) | … | 0.1926(0.1838,0.2014) | 0.2286(0.2197,0.2375) |
|  | **Visit impacts** | 0.1265(0.1200,0.1330) | 0.1090(0.1018,0.1161) | … | 0.0339(0.0251,0.0427) | 0.0021(-0.006,0.0110) |
| **West** | **Predicted OEH** | 0.1903(0.1850,0.1956) | 0.2161(0.2107,0.2216) | … | 0.2008(0.1952,0.2065) | 0.2335(0.2279,0.2392) |
|  | **Visit impacts** | 0.0985(0.0932,0.1038) | 0.0603(0.0548,0.0657) | … | 0.0152(0.0096,0.0209) | 0.0111(0.0054,0.0167) |

Note: Predicted OEH: the predicted value with 95% CI of OEH visits per capita after the pandemic; Visit impacts: estimated impacts with 95% CI of OEH visits per capita after the pandemic with the respective 95% CI. Due to space constraints, we only showed data for some months after the epidemic.

**Table R3. Estimated absolute impacts for provinces of mainland China by months.**

| **Province** | **Date** | **2020/02** | **2020/03** |  | **2021/02** | **2021/03** |
| --- | --- | --- | --- | --- | --- | --- |
| **Anhui** | **Predicted OEH** | 0.1547(0.1456,0.1638) | 0.1812(0.1719,0.1904) | … | 0.1644(0.1499,0.1788) | 0.1994(0.1849,0.2140) |
|  | **Visit impacts** | 0.0743(0.0652,0.0834) | 0.0418(0.0326,0.0511) | … | -0.012(-0.027,0.0015) | 0.0088(-0.005,0.0233) |
| **Beijing** | **Predicted OEH** | 0.4856(0.4568,0.5143) | 0.6518(0.6224,0.6811) | … | 0.5014(0.4712,0.5315) | 0.6839(0.6537,0.7141) |
|  | **Visit impacts** | 0.3306(0.3019,0.3593) | 0.4479(0.4186,0.4773) | … | 0.0908(0.0606,0.1209) | 0.1004(0.0702,0.1306) |
| **Chongqing** | **Predicted OEH** | 0.1897(0.1833,0.1961) | 0.2232(0.2168,0.2296) | … | 0.2025(0.1960,0.2090) | 0.2358(0.2293,0.2423) |
|  | **Visit impacts** | 0.1007(0.0943,0.1072) | 0.0694(0.0630,0.0759) | … | 0.0023(-0.004,0.0088) | 0.0019(-0.004,0.0084) |
| **Fujian** | **Predicted OEH** | 0.1567(0.1490,0.1644) | 0.2167(0.2080,0.2253) | … | 0.1574(0.1470,0.1678) | 0.2149(0.2043,0.2254) |
|  | **Visit impacts** | 0.0630(0.0552,0.0707) | 0.0589(0.0502,0.0675) | … | -0.011(-0.022,-0.001) | -0.011(-0.022,-0.001) |
| **Gansu** | **Predicted OEH** | 0.1713(0.1643,0.1783) | 0.1917(0.1847,0.1988) | … | 0.1940(0.1865,0.2016) | 0.2196(0.2121,0.2272) |
|  | **Visit impacts** | 0.0839(0.0769,0.0909) | 0.0388(0.0317,0.0459) | … | 0.0323(0.0247,0.0398) | 0.0238(0.0162,0.0313) |
| **Guangdong** | **Predicted OEH** | 0.2023(0.1899,0.2146) | 0.2933(0.2809,0.3056) | … | 0.2114(0.1990,0.2239) | 0.3015(0.2890,0.3140) |
|  | **Visit impacts** | 0.1052(0.0928,0.1175) | 0.1315(0.1191,0.1439) | … | 0.0016(-0.010,0.0141) | 0.0362(0.0238,0.0487) |
| **Guangxi** | **Predicted OEH** | 0.1661(0.1599,0.1723) | 0.1992(0.1929,0.2054) | … | 0.1717(0.1652,0.1782) | 0.2072(0.2007,0.2137) |
|  | **Visit impacts** | 0.0809(0.0747,0.0871) | 0.0631(0.0569,0.0694) | … | 0.0034(-0.003,0.0099) | 0.0111(0.0046,0.0176) |
| **Guizhou** | **Predicted OEH** | 0.1460(0.1419,0.1502) | 0.1747(0.1705,0.1788) | … | 0.1569(0.1518,0.1619) | 0.1878(0.1827,0.1928) |
|  | **Visit impacts** | 0.0566(0.0525,0.0608) | 0.0404(0.0362,0.0446) | … | -0.006(-0.011,-0.001) | 0.0019(-0.003,0.0069) |
| **Hainan** | **Predicted OEH** | 0.1702(0.1609,0.1795) | 0.1855(0.1761,0.1950) | … | 0.1383(0.1187,0.1578) | 0.2056(0.1858,0.2255) |
|  | **Visit impacts** | 0.1005(0.0912,0.1098) | 0.0579(0.0484,0.0673) | … | -0.019(-0.039,-0.000) | 0.0065(-0.013,0.0264) |
| **Hebei** | **Predicted OEH** | 0.1532(0.1476,0.1588) | 0.1830(0.1773,0.1886) | … | 0.1566(0.1480,0.1651) | 0.1852(0.1766,0.1938) |
|  | **Visit impacts** | 0.0729(0.0673,0.0785) | 0.0506(0.0449,0.0562) | … | -0.030(-0.039,-0.022) | -0.034(-0.042,-0.025) |
| **Heilongjiang** | **Predicted OEH** | 0.1799(0.1750,0.1847) | 0.2141(0.2078,0.2204) | … | 0.1507(0.1345,0.1670) | 0.1778(0.1589,0.1967) |
|  | **Visit impacts** | 0.1345(0.1297,0.1394) | 0.1213(0.1150,0.1276) | … | 0.0226(0.0063,0.0388) | -0.026(-0.045,-0.007) |
| **Henan** | **Predicted OEH** | 0.1971(0.1921,0.2022) | 0.2079(0.2028,0.2130) | … | 0.2111(0.2057,0.2166) | 0.2339(0.2284,0.2393) |
|  | **Visit impacts** | 0.1167(0.1116,0.1218) | 0.0687(0.0636,0.0738) | … | 0.0350(0.0295,0.0404) | 0.0327(0.0273,0.0382) |
| **Hubei** | **Predicted OEH** | 0.1585(0.1502,0.1669) | 0.2165(0.2081,0.2249) | … | 0.1354(0.1230,0.1479) | 0.1876(0.1751,0.2001) |
|  | **Visit impacts** | 0.1585(0.1502,0.1669) | 0.1330(0.1246,0.1414) | … | -0.029(-0.042,-0.017) | -0.028(-0.040,-0.015) |
| **Hunan** | **Predicted OEH** | 0.1328(0.1282,0.1374) | 0.1464(0.1418,0.1510) | … | 0.1306(0.1237,0.1374) | 0.1537(0.1467,0.1607) |
|  | **Visit impacts** | 0.0729(0.0683,0.0775) | 0.0450(0.0403,0.0496) | … | 0.0035(-0.003,0.0104) | -0.000(-0.007,0.0065) |
| **Jiangsu** | **Predicted OEH** | 0.2371(0.2246,0.2496) | 0.2789(0.2630,0.2949) | … | 0.2282(0.2114,0.2450) | 0.2829(0.2658,0.2999) |
|  | **Visit impacts** | 0.1273(0.1148,0.1398) | 0.0994(0.0834,0.1154) | … | 0.0034(-0.013,0.0202) | 0.0027(-0.014,0.0197) |
| **Jiangxi** | **Predicted OEH** | 0.1434(0.1388,0.1480) | 0.1594(0.1548,0.1640) | … | 0.1572(0.1521,0.1623) | 0.1776(0.1725,0.1827) |
|  | **Visit impacts** | 0.0748(0.0702,0.0794) | 0.0453(0.0407,0.0499) | … | 0.0134(0.0083,0.0185) | 0.0142(0.0091,0.0193) |
| **Jilin** | **Predicted OEH** | 0.1924(0.1848,0.1999) | 0.2281(0.2205,0.2357) | … | 0.2097(0.2017,0.2177) | 0.2477(0.2397,0.2557) |
|  | **Visit impacts** | 0.1157(0.1081,0.1233) | 0.0911(0.0835,0.0986) | … | 0.0383(0.0303,0.0463) | 0.0176(0.0095,0.0256) |
| **Liaoning** | **Predicted OEH** | 0.1835(0.1760,0.1910) | 0.2130(0.2056,0.2205) | … | 0.0776(0.0430,0.1122) | 0.1100(0.0712,0.1488) |
|  | **Visit impacts** | 0.0973(0.0899,0.1048) | 0.0713(0.0638,0.0788) | … | -0.096(-0.131,-0.061) | -0.130(-0.169,-0.091) |
| **Nei Mongol** | **Predicted OEH** | 0.1856(0.1765,0.1948) | 0.2298(0.2207,0.2389) | … | 0.1986(0.1889,0.2083) | 0.2460(0.2363,0.2557) |
|  | **Visit impacts** | 0.1164(0.1073,0.1255) | 0.0838(0.0747,0.0929) | … | 0.0270(0.0173,0.0367) | 0.0033(-0.006,0.0130) |
| **Ningxia** | **Predicted OEH** | 0.2044(0.1946,0.2142) | 0.2983(0.2883,0.3082) | … | 0.2375(0.2237,0.2513) | 0.2833(0.2695,0.2971) |
|  | **Visit impacts** | 0.1014(0.0916,0.1112) | 0.1080(0.0981,0.1180) | … | 0.0144(0.0006,0.0282) | -0.000(-0.014,0.0133) |
| **Qinghai** | **Predicted OEH** | 0.1624(0.1430,0.1819) | 0.2111(0.1886,0.2336) | … | 0.1941(0.1659,0.2223) | 0.2286(0.2001,0.2572) |
|  | **Visit impacts** | 0.0465(0.0270,0.0659) | 0.0212(-0.001,0.0437) | … | 0.0108(-0.017,0.0391) | 0.0007(-0.027,0.0293) |
| **Shaanxi** | **Predicted OEH** | 0.2122(0.2047,0.2197) | 0.2361(0.2284,0.2437) | … | 0.2152(0.2063,0.2240) | 0.2495(0.2405,0.2585) |
|  | **Visit impacts** | 0.1387(0.1312,0.1462) | 0.0873(0.0797,0.0949) | … | 0.0306(0.0217,0.0394) | 0.0185(0.0095,0.0275) |
| **Shandong** | **Predicted OEH** | 0.1784(0.1710,0.1857) | 0.2075(0.2001,0.2149) | … | 0.1749(0.1634,0.1863) | 0.2028(0.1913,0.2143) |
|  | **Visit impacts** | 0.0863(0.0790,0.0936) | 0.0647(0.0573,0.0721) | … | -0.004(-0.016,0.0066) | -0.017(-0.029,-0.006) |
| **Shanghai** | **Predicted OEH** | 0.4669(0.4338,0.5000) | 0.6123(0.5787,0.6459) | … | 0.4475(0.4132,0.4818) | 0.6203(0.5860,0.6546) |
|  | **Visit impacts** | 0.2803(0.2472,0.3135) | 0.3063(0.2727,0.3400) | … | 0.0279(-0.006,0.0622) | 0.0609(0.0266,0.0952) |
| **Shanxi** | **Predicted OEH** | 0.1193(0.1131,0.1256) | 0.1540(0.1473,0.1606) | … | 0.1109(0.0985,0.1233) | 0.1447(0.1316,0.1578) |
|  | **Visit impacts** | 0.0503(0.0441,0.0566) | 0.0273(0.0207,0.0340) | … | -0.031(-0.043,-0.018) | -0.047(-0.060,-0.034) |
| **Sichuan** | **Predicted OEH** | 0.2146(0.2078,0.2215) | 0.2434(0.2365,0.2504) | … | 0.2309(0.2237,0.2381) | 0.2652(0.2579,0.2724) |
|  | **Visit impacts** | 0.1077(0.1009,0.1146) | 0.0742(0.0673,0.0812) | … | 0.0256(0.0184,0.0328) | 0.0174(0.0102,0.0246) |
| **Tianjin** | **Predicted OEH** | 0.3431(0.3207,0.3655) | 0.4634(0.4403,0.4865) | … | 0.3600(0.3273,0.3927) | 0.4812(0.4480,0.5143) |
|  | **Visit impacts** | 0.2049(0.1824,0.2273) | 0.2477(0.2246,0.2708) | … | 0.0407(0.0080,0.0734) | 0.0653(0.0321,0.0984) |
| **Tibet** | **Predicted OEH** | - | - | … | - | - |
|  | **Visit impacts** | - | - | … | - | - |
| **Xinjiang** | **Predicted OEH** | 0.1388(0.1309,0.1468) | 0.1643(0.1560,0.1726) | … | 0.1641(0.1534,0.1749) | 0.1641(0.1534,0.1749) |
|  | **Visit impacts** | 0.0760(0.0680,0.0840) | -0.000(-0.008,0.0080) | … | -0.009(-0.019,0.0016) | -0.047(-0.058,-0.037) |
| **Yunnan** | **Predicted OEH** | 0.1902(0.1853,0.1951) | 0.2181(0.2123,0.2239) | … | 0.2004(0.1929,0.2078) | 0.2246(0.2170,0.2322) |
|  | **Visit impacts** | 0.0743(0.0694,0.0793) | 0.0439(0.0381,0.0497) | … | -0.002(-0.009,0.0052) | -0.002(-0.009,0.0055) |
| **Zhejiang** | **Predicted OEH** | 0.3498(0.3353,0.3643) | 0.3982(0.3837,0.4127) | … | 0.3111(0.2950,0.3272) | 0.3825(0.3664,0.3986) |
|  | **Visit impacts** | 0.2105(0.1959,0.2250) | 0.1464(0.1319,0.1609) | … | -0.005(-0.021,0.0103) | 0.0027(-0.013,0.0188) |

Note: Predicted OEH: the predicted value with 95% CI of OEH visits per capita after the pandemic; Visit impacts: estimated impacts with 95% CI of OEH visits per capita after the pandemic with the respective 95% CI. -: data are unavailable.

**Q4.** It is hard to read the figures. Please, improve the quality of all five figures.

Response: Thanks for the suggestion. Firstly, in the original manuscript, we had provided figures with a resolution of 300 ppi, which met the journal's requirements for figure clarity in publications. However, in the submission system, our figures were compressed, resulting in poor figure quality. The original figures can be downloaded via the submission system. Secondly, to make the figures clearer, in the revised manuscript, we adjusted the layout of the **Figure 1**, **2**, and **3** in the revised manuscript.

**Responses to reviewer 2:**
**Q1**. There seems to be a potential issue with model over-fitting. The authors estimated separate S-ARIMA models for each Chinese province; each model has a different time series signature. These can be quite different, with between 1 and 3 autoregressive lags, integration, etc. Because these time series were selected with BIC, it is almost certain they will overfit the data. If the aim was predictive accuracy at the province level, that would probably be fine, but since the aim is inter-region comparison, it is difficult to compare the counterfactual estimates from ARIMA models with different signatures. I might suggest the authors use a simple AR(1,1,0) model, which seems like it would fit the data, and apply it to all provinces. Or perhaps use the modal AR signature of all models, and then apply that to all provinces.

Response: We appreciate the valuable comments. First of all, we want to state that the predictive accuracy at the province level was as important as inter-region comparison in our study. The inter-region comparison was based on accurately estimating the impact of COVID-19 pandemic on health service utilization in each region. Furthermore, there were remarkable differences in OEH visit per capita among provinces/regions. For example, the OEH series of Anhui in **Figure R3a1** below grew steadily, while the OEH series of Guangdong in **Figure R3b1** below stayed basically at the same level. Therefore, data for all provinces cannot be predicted using a unique model, e.g., AR (1,1,0) model.

We have taken Anhui and Guangdong provinces as an example and analyzed our data by using the suggested AR (1,1,0) model and the current optimal S-ARIMA model separately. The results are shown in **Figure R3** below and it is clear that the current model can better reflect the OEH series, better forecast the post-pandemic OEH series, and then make a more accurate assessment of the impact of the pandemic.

**Figure R3. Comparison between the forecast of current model and AR (1,1,0) model for Anhui (left) and Guangdong (right).**

**Q2**. In any case, while the model allows the authors to predict counterfactual trends with very high accuracy, the aim of the paper seems to be to infer different counterfactual gaps from policies and demographic factors. It would be better if these factors, such as the age structure of the province, were directly incorporated into the model as exogenous (non-varying) factors. Similarly, the total counterfactual drop can be calculated by using a dummy for post-Jan 2020 and reporting the coefficient. If the aim is simply to do regional comparison, then using a pre/post cross-sectional model might even be preferred as it would allow for the authors to test directly their claims regarding age structure and its association with hospital visits.

Response: We appreciated the reviewer for the critical comments. However, the purpose of our study was not to infer different counterfactual gaps from policies and demographic factors, such as policies of COVID-19 epidemic prevention and control, and age structure. As we mentioned in the response to Q1 of reviewer 2, the primary aim of this study was to predict the impact of COVID-19 pandemic on health service utilization in each province/region accurately.

Moreover, since the social polies were always adjusted during the pandemic, there were no specific quantitative indicators to measure such changing policies, and only qualitative descriptions were existed. Additionally, although the aging of China is deepening, the population aging is a very slow process and the monthly aging rate (percentage over 60 years old) in each province in China was almost a constant series from 2016/01 to 2021/03. Therefore, it does not make sense to include this factor into an ARIMAX model as an exogenous factor.

Policies and demographic factors were only included in the Discussion Section as possible explanations and we also added one limitation to state reasons for not including these factors in S-ARIMA models (page 18-19, line 402-407).

**Figure R4. Aging rates in different provinces in 2016 and 2020.**

Note: Figure R4 shows the proportion of people aged over 60 years old in 31 provinces of mainland China in 2016 and 2020, respectively. Source: The percentage of older adults in 2020 (right column) is exactly obtained from the seventh population census ^[4]^. Due to lack of data, the percentage of older adults in 2016 (left column) is estimated by smooth processing based on the sixth and seventh population census^[4, 5]^.

**Q3**. Part of the above is for the authors to be more clear about the purpose of their paper. The introduction is framed as identifying where hospital systems are less resilient, but then the discussion and the conclusion are much more about the effect of age demographics, etc. If the aim is to test these hypotheses, these should be formally incorporated and addressed in the paper’s research design section.

Response: We thank the reviewer for pointing out the need for clarification and are sorry for not making the research aim and hypotheses clear in our manuscript. We have added the research aim and hypotheses in the Introduction section (page 3, line 60-66; page 4, line 80-87) and revised the whole manuscript, especially the Discussion section, correspondingly (page 14-15, line 317-324).

**Q4**. The reported effects are in per capita visits, but it would seem the relevant metric should be percentage decrease from pre-COVID baseline. Otherwise, areas with high per capita visits pre-COVID, like Beijing, will show more dramatic falls because there were more people who went to the hospital in baseline. Also, for these top line results, CIs should be included wherever possible to show uncertainty in these impacts.

Response: We thank the reviewer for providing this helpful comment, which we also think is a very important aspect of the impact. We have done complementary analyses of the relative impacts of the pandemic and represented results in the revised manuscript (page 10, line 213-221; page 12, line 249-254; **Table 1**; **Figure 2b,3b**).

In the complementary analyses, the definition of the baseline was an important issue, and there is no standard definition. We chose the mean OEH visits per capita 1 month, 3 months, 6 months, 1 year, and 4 years before the pandemic as the baseline, respectively, and conducted five different analyses (**Table R4** below). As **Table R4** below shows, the baselines were generally consistent across different definitions. In the revised manuscript, we chose the mean OEH visits per capita one year before the pandemic as the baseline. The relative instant impacts and the relative accumulative impacts are listed in the following **Table R5**, **Figure R5** and **R7**. It should be noted that, whichever of the baseline we choose, the qualitative results were consistent with each other (**Figure R6 and R8** below).

**Table R4. Five different baselines of OEH visits per capita in different provinces and regions in mainland China.**

| **Province/Region** | | | **The mean OEH visits per capita in the** | | | | |
| --- | --- | --- | --- | --- | --- | --- | --- |
|  |  |  | **Last 1 month** | **Last 3 months** | **Last 6 months** | **The past one year** | **The past four years** |
| China | | | 0.1901 | 0.2126 | 0.2202 | 0.2182 | 0.2055 |
|  | Central | | 0.1264 | 0.1541 | 0.1634 | 0.1648 | 0.1512 |
|  |  | Anhui | 0.1741 | 0.1546 | 0.1560 | 0.1551 | 0.1401 |
|  |  | Henan | 0.1696 | 0.1824 | 0.1881 | 0.1863 | 0.1689 |
|  |  | Hubei | 0.1921 | 0.2086 | 0.2085 | 0.2020 | 0.1851 |
|  |  | Hunan | 0.1213 | 0.1325 | 0.1396 | 0.1423 | 0.1301 |
|  |  | Jiangxi | 0.1350 | 0.1439 | 0.1461 | 0.1481 | 0.1300 |
|  |  | Shanxi | 0.1271 | 0.1430 | 0.1522 | 0.1498 | 0.1391 |
|  | East | | 0.2412 | 0.2696 | 0.2785 | 0.2737 | 0.2657 |
|  |  | Beijing | 0.4495 | 0.5507 | 0.5794 | 0.5717 | 0.5760 |
|  |  | Fujian | 0.1813 | 0.1966 | 0.2030 | 0.2018 | 0.1982 |
|  |  | Guangdong | 0.2182 | 0.2531 | 0.2637 | 0.2640 | 0.2598 |
|  |  | Hainan | 0.1569 | 0.1613 | 0.1711 | 0.1743 | 0.1574 |
|  |  | Hebei | 0.1660 | 0.1751 | 0.1761 | 0.1687 | 0.1528 |
|  |  | Jiangsu | 0.2562 | 0.2691 | 0.2710 | 0.2508 | 0.2547 |
|  |  | Shandong | 0.1872 | 0.1982 | 0.2045 | 0.2005 | 0.1858 |
|  |  | Shanghai | 0.4550 | 0.5249 | 0.5504 | 0.5600 | 0.5330 |
|  |  | Tianjin | 0.3269 | 0.4031 | 0.4202 | 0.4169 | 0.4295 |
|  |  | Zhejiang | 0.3186 | 0.3661 | 0.3815 | 0.3807 | 0.3689 |
|  | Northeast | | 0.1634 | 0.1813 | 0.1904 | 0.1913 | 0.1792 |
|  |  | Heilongjiang | 0.1460 | 0.1620 | 0.1689 | 0.1708 | 0.1612 |
|  |  | Jilin | 0.1661 | 0.1825 | 0.1923 | 0.1926 | 0.1769 |
|  |  | Liaoning | 0.1750 | 0.1952 | 0.2057 | 0.2060 | 0.1946 |
|  | West | | 0.1825 | 0.1929 | 0.1966 | 0.1949 | 0.1771 |
|  |  | Chongqing | 0.1854 | 0.2006 | 0.2044 | 0.2036 | 0.1856 |
|  |  | Gansu | 0.1687 | 0.1658 | 0.1662 | 0.1664 | 0.1544 |
|  |  | Guangxi | 0.1612 | 0.1767 | 0.1826 | 0.1824 | 0.1710 |
|  |  | Guizhou | 0.1573 | 0.1629 | 0.1651 | 0.1646 | 0.1484 |
|  |  | Nei Mongol | 0.1593 | 0.1767 | 0.1858 | 0.1887 | 0.1725 |
|  |  | Ningxia | 0.2241 | 0.2448 | 0.2581 | 0.2502 | 0.2391 |
|  |  | Qinghai | 0.1749 | 0.1901 | 0.1967 | 0.1902 | 0.1753 |
|  |  | Shaanxi | 0.1823 | 0.2035 | 0.2085 | 0.2067 | 0.1861 |
|  |  | Sichuan | 0.2002 | 0.2157 | 0.2185 | 0.2166 | 0.1923 |
|  |  | Tibet | - | - | - | - | - |
|  |  | Xinjiang | 0.1614 | 0.1646 | 0.1703 | 0.1697 | 0.1646 |
|  |  | Yunnan | 0.2049 | 0.2127 | 0.2115 | 0.2072 | 0.1885 |

**Note** -: data are unavailable.

**Figure R5. Relative instant impact (%) based on the mean OEH visits per capita one year before the pandemic.**

**Figure R6. Relative instant impact (%) based on other baseline definitions.**

Panel a) b) c) and d) shows maps of relative instant impacts in mainland China based on four types of baselines (mean OEH visits per capita 1 month, 3months, 6months, and 4 years before the pandemic), respectively.

**Figure R7. Relative accumulative impact (%) based on baseline as the mean OEH visits per capita one year before the pandemic.**

**Figure R8. Relative accumulative impact (%) based on other baseline definitions.**

Panel a) b) c) and d) shows maps of relative accumulative impacts in mainland China based on four types of baselines (mean OEH visits per capita 1 month, 3months, 6months, and 4 years before the pandemic), respectively.

In order to fully reflect the various impacts of the pandemic on the volume of OEH visits per capita, we revised **Table 1** in the manuscript by adding two columns of relative impacts (namely **Table R5** in this response letter).

**Table R5. Impacts of OEH visits per capita due to COVID-19.**

| **Region/Province** | | | **Instant impacts** | | |  | **Accumulative impacts** | | |
| --- | --- | --- | --- | --- | --- | --- | --- | --- | --- |
|  |  |  | **Absolute** |  | **Relative (%)** |  | **Absolute** |  | **Relative (%)** |
| China | | | 0.1256 (0.1187,0.1325) |  | 57.59 (54.42,60.75) |  | 0.4676 |  | 16.48 |
|  | Central | | 0.1279 (0.1220,0.1337) |  | 77.63 (74.08,81.18) |  | 0.3324 |  | 15.52 |
|  |  | Anhui | 0.0743 (0.0652,0.0834) |  | 47.94 (42.08,53.80) |  | 0.0173 |  | 0.86 |
|  |  | Henan | 0.1167 (0.1116,0.1218) |  | 62.66 (59.93,65.39) |  | 0.5088 |  | 21.01 |
|  |  | Hubei | 0.1585 (0.1502,0.1669) |  | 78.50 (74.35,82.64) |  | 0.5632 |  | 19.91 |
|  |  | Hunan | 0.0729 (0.0683,0.0775) |  | 51.23 (48.00,54.46) |  | 0.1440 |  | 7.79 |
|  |  | Jiangxi | 0.0748 (0.0702,0.0794) |  | 50.54 (47.44,53.64) |  | 0.2615 |  | 13.58 |
|  |  | Shanxi | 0.0503 (0.0441,0.0566) |  | 33.62 (29.44,37.80) |  | -0.1674 |  | -8.59 |
|  | East | | 0.1215 (0.1126,0.1303) |  | 44.39 (41.16,47.61) |  | 0.4459 |  | 12.53 |
|  |  | Beijing | 0.3306 (0.3019,0.3593) |  | 57.83 (52.80,62.85) |  | 2.5187 |  | 33.89 |
|  |  | Fujian | 0.0630 (0.0552,0.0707) |  | 31.23 (27.39,35.06) |  | 0.1394 |  | 5.31 |
|  |  | Guangdong | 0.1052 (0.0928,0.1175) |  | 39.86 (35.18,44.54) |  | 0.5939 |  | 17.31 |
|  |  | Hainan | 0.1005 (0.0912,0.1098) |  | 57.68 (52.34,63.02) |  | 0.2292 |  | 10.12 |
|  |  | Hebei | 0.0729 (0.0673,0.0785) |  | 43.23 (39.92,46.54) |  | 0.0820 |  | 3.74 |
|  |  | Jiangsu | 0.1273 (0.1148,0.1398) |  | 50.79 (45.81,55.77) |  | 0.3684 |  | 11.30 |
|  |  | Shandong | 0.0863 (0.0790,0.0936) |  | 43.06 (39.41,46.71) |  | 0.1981 |  | 7.60 |
|  |  | Shanghai | 0.2803 (0.2472,0.3135) |  | 50.06 (44.15,55.98) |  | 1.3501 |  | 18.54 |
|  |  | Tianjin | 0.2049 (0.1824,0.2273) |  | 49.15 (43.77,54.53) |  | 1.2867 |  | 23.74 |
|  |  | Zhejiang | 0.2105 (0.1959,0.2250) |  | 55.29 (51.47,59.10) |  | 0.4585 |  | 9.26 |
|  | Northeast | | 0.1265 (0.1200,0.1330) |  | 66.16 (62.78,69.55) |  | 0.5898 |  | 23.72 |
|  |  | Heilongjiang | 0.1345 (0.1297,0.1394) |  | 78.78 (75.94,81.62) |  | 0.6217 |  | 27.99 |
|  |  | Jilin | 0.1157 (0.1081,0.1233) |  | 60.07 (56.14,64.01) |  | 0.6400 |  | 25.56 |
|  |  | Liaoning | 0.0973 (0.0899,0.1048) |  | 47.27 (43.64,50.89) |  | -0.4188 |  | -15.64 |
|  | West | | 0.0985 (0.0932,0.1038) |  | 50.57 (47.85,53.28) |  | 0.3523 |  | 13.91 |
|  |  | Chongqing | 0.1007 (0.0943,0.1072) |  | 49.50 (46.34,52.65) |  | 0.2767 |  | 10.45 |
|  |  | Gansu | 0.0839 (0.0769,0.0909) |  | 50.46 (46.23,54.68) |  | 0.4372 |  | 20.21 |
|  |  | Guangxi | 0.0809 (0.0747,0.0871) |  | 44.36 (40.95,47.77) |  | 0.3213 |  | 13.55 |
|  |  | Guizhou | 0.0566 (0.0525,0.0608) |  | 34.44 (31.90,36.98) |  | 0.1444 |  | 6.75 |
|  |  | Nei Mongol | 0.1164 (0.1073,0.1255) |  | 61.69 (56.86,66.53) |  | 0.4067 |  | 16.58 |
|  |  | Ningxia | 0.1014 (0.0916,0.1112) |  | 40.52 (36.61,44.44) |  | 0.3345 |  | 10.28 |
|  |  | Qinghai | 0.0465 (0.0270,0.0659) |  | 24.45 (14.22,34.68) |  | 0.0707 |  | 2.86 |
|  |  | Shaanxi | 0.1387 (0.1312,0.1462) |  | 67.13 (63.51,70.76) |  | 0.4754 |  | 17.69 |
|  |  | Sichuan | 0.1077 (0.1009,0.1146) |  | 49.76 (46.6,52.92) |  | 0.4704 |  | 16.71 |
|  |  | Tibet | - |  | - |  | - |  | - |
|  |  | Xinjiang | 0.0760 (0.0680,0.0840) |  | 44.80 (40.11,49.50) |  | -0.0162 |  | -0.74 |
|  |  | Yunnan | 0.0743 (0.0694,0.0793) |  | 35.88 (33.50,38.27) |  | 0.2181 |  | 8.10 |

Note: The 95% confidence intervals of instant impacts in each province and region were presented in the brackets of the 2nd and 3rd column and there were no interval estimations for accumulative impacts. -: No prediction and no data.

**Q5**. The impact on per capita visits plot should be a heatmap, where the colors of the provinces are the amount of drop in visits (Figure 2).

Response: Thanks for your suggestions, the current manuscript version has been revised (**Figure 2a**; **Figure 3a**). The modified heatmap is demonstrated below (**Figure R9**).

**Figure R9. Instant impacts of COVID-19 on OEH visits per capita in mainland China.**

Again, I would like to express our great appreciation to you and reviewers for the critical comments on our paper.

Best regards.

Yours sincerely,

Wen Chen, Professor, Ph.D.

Department of Medical Statistics, School of Public Health, Sun Yat-sen University,

Email: chenw43@mail.sysu.edu.cn.

**Reference**

1.	National Bureau of Statistics of China. China Statistical Yearbook 2010: China Statistics Press; 2010.

2.	National Bureau of Statistics of China. China Statistical Yearbook 2020: China Statistics Press; 2020.

3.	National Health Commission of China. China Health Statistics Yearbook 2020. Beijing: Peking Union Medical College Press; 2020.

4.	Office of the Leading Group of the State Council for the Seventh National Population Census. China Population Census Yearbook 2020: China Statistics Press; 2021.

5.	Population Census Office under the State Council, Department of Population and Employment Statistics National Bureau of Statistics. Tabulation on the 2010 Population Census of the People's Republic of China: China Statistics Press; 2012.
